# Supplementary figures and images for: The role and targeting potential analysis of angiogenesis-related target THY1 in DSS-induced acute colitis in mice
Source: PLoS One. 2026 Jun 2;21(6):e0350385. doi: 10.1371/journal.pone.0350385 (PMC13229366; doi:10.1371/journal.pone.0350385)

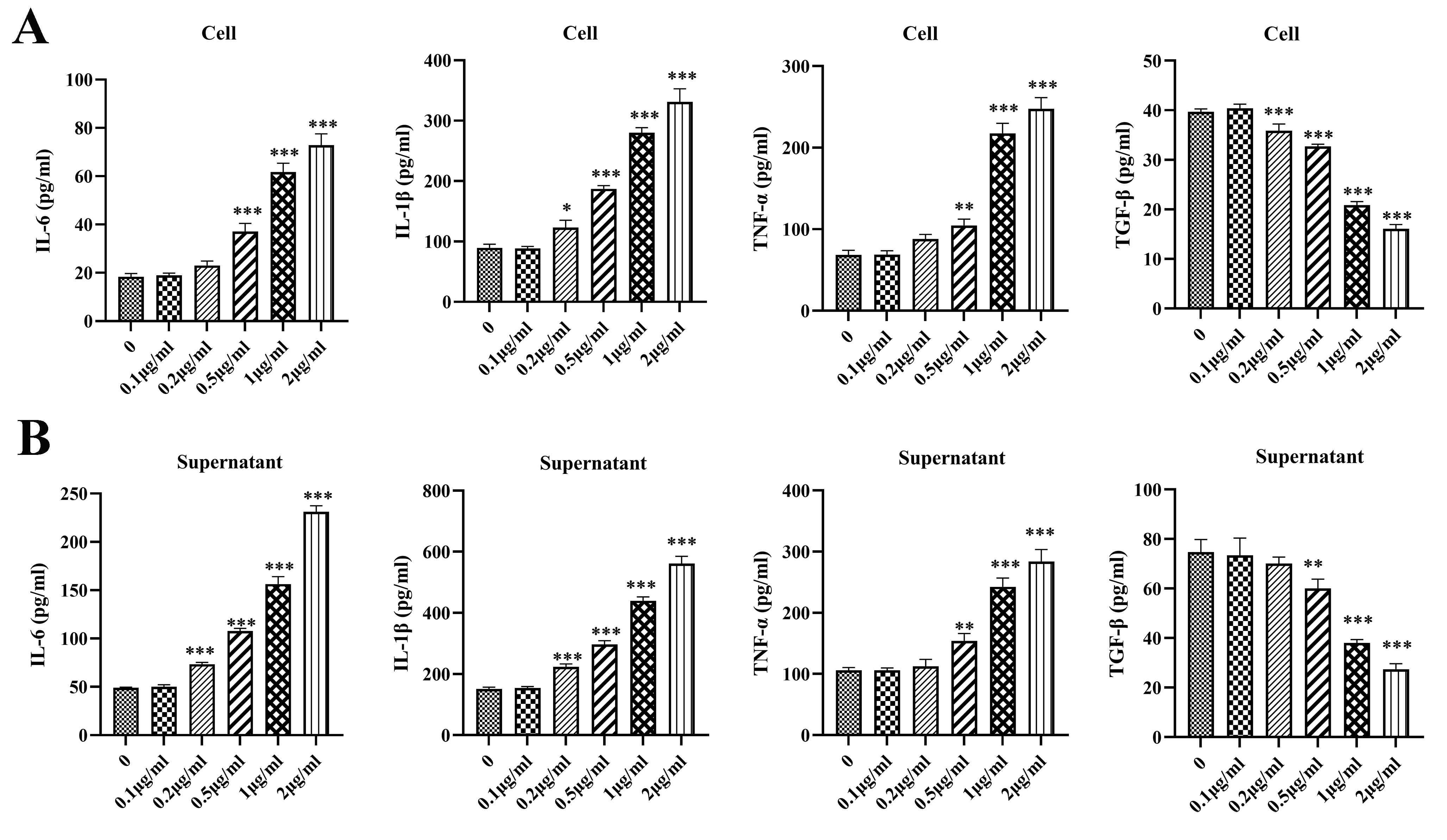

Supplement: S1 Fig — The expression levels of inflammatory factors including IL-1β, IL-6, TNF-α, and TGF-β in NCM460 cells (A) and its supernatant (B) were detected by ELISA. NCM460 cells were gradient intervention with different concentrations of DSS (e.g., 0, 0.1, 0.2, 0.5, 1, 2 μg/mL) for 12 hours. A minimum of three separate experiments were carried out and the data presented are expressed as the mean ± SD. *P < 0.05, **P < 0.01, ***P < 0.001. (TIF) [file pone.0350385.s001.tif]

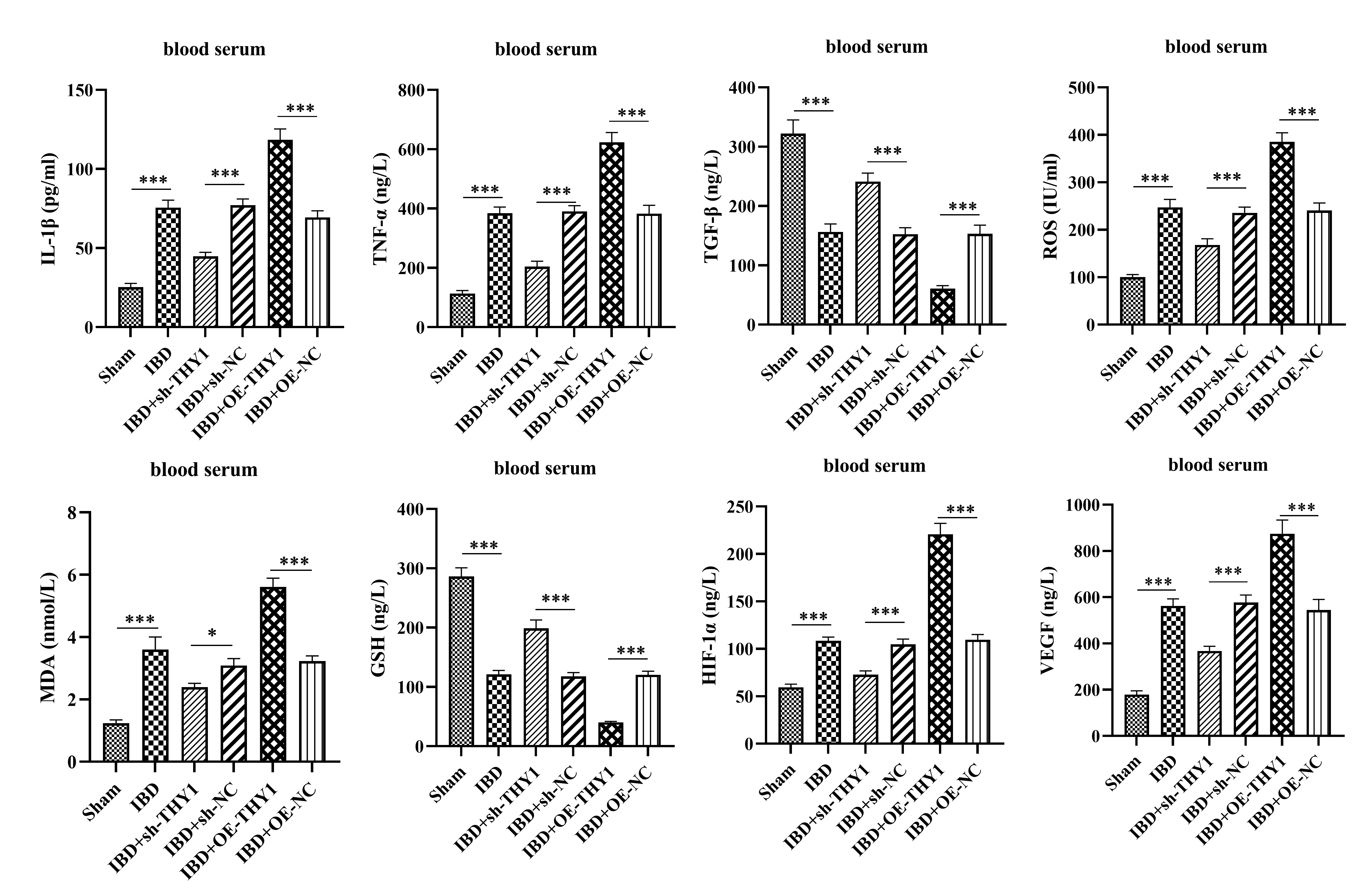

Supplement: S2 Fig — The expression levels of inflammatory factors (IL-1β, TNF-α and TGF-β), oxidative stress-related molecules (ROS, MDA and GSH) and angiogenesis-related stimulatory factors (HIF-1α and VEGF) in mouse blood serum samples of different treatment groups were detected by enzyme-linked immunosorbent assay (ELISA). A minimum of three separate experiments were carried out and the data presented are expressed as the mean ± SD. *P < 0.05, **P < 0.01, ***P < 0.001. (TIF) [file pone.0350385.s002.tif]
