# Supplementary material for: The role and targeting potential analysis of angiogenesis-related target THY1 in DSS-induced acute colitis in mice
Source: PLoS One. 2026 Jun 2;21(6):e0350385. doi: 10.1371/journal.pone.0350385 (PMC13229366; doi:10.1371/journal.pone.0350385)
Supplement: S3 Fig — (PDF) [file pone.0350385.s003.pdf]

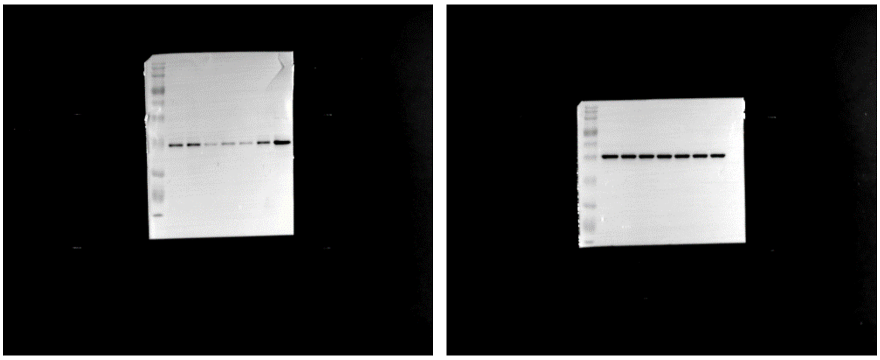

The full uncropped Gels and Blots images of THY1 and β-actin for Fig1A

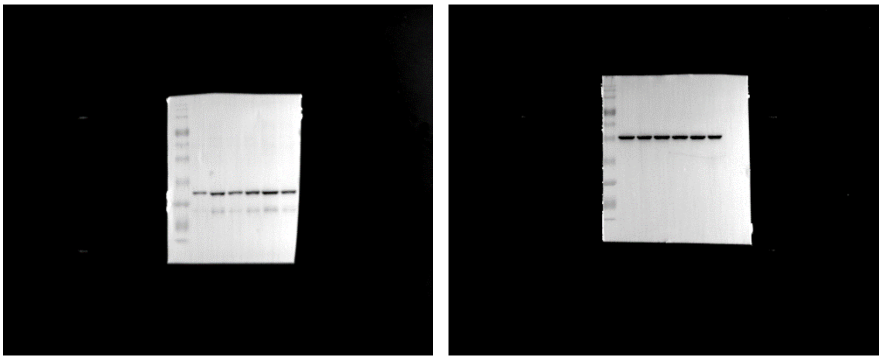

The full uncropped Gels and Blots images of THY1 and β-actin for Fig 1B

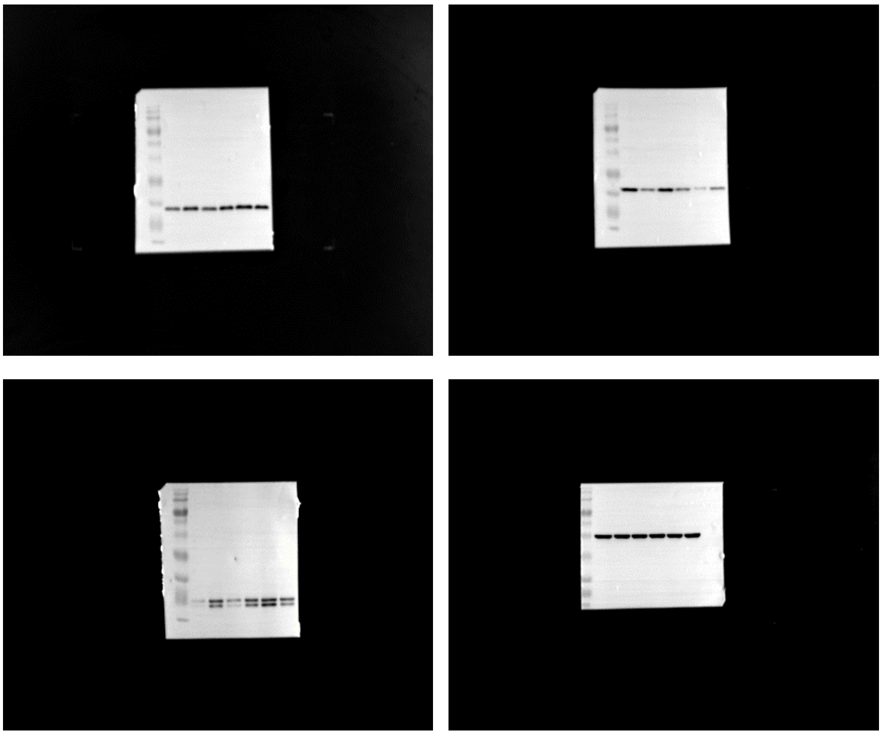

The full uncropped Gels and Blots images of Bax, Bcl-2, Cleaved caspase-3 and β-actin for Fig 3B

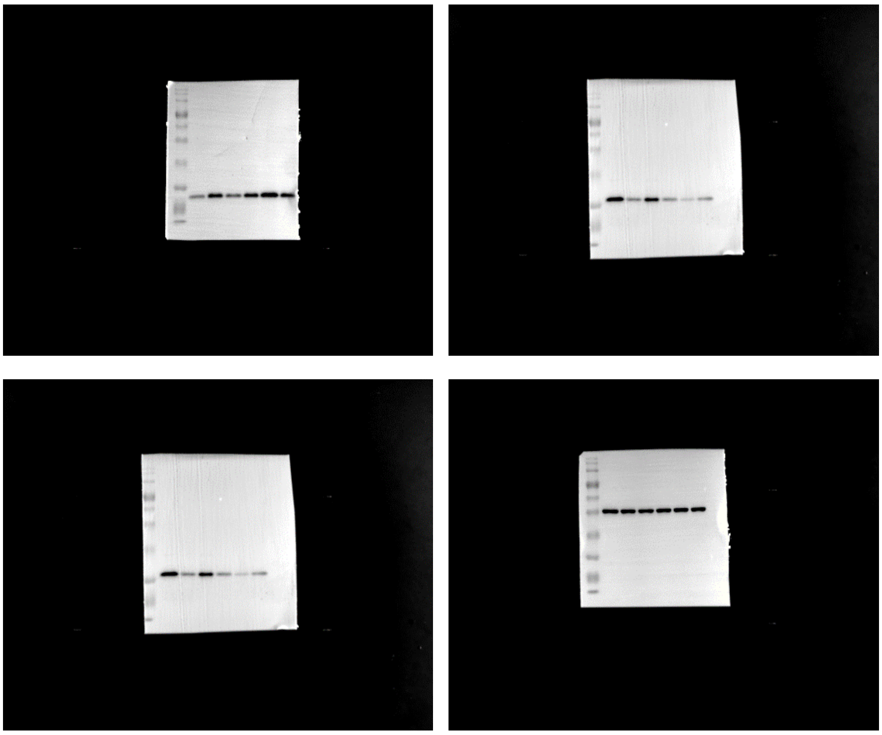

The full uncropped Gels and Blots images of Bax, Bcl-2, Cleaved caspase-3 and β-actin for Fig 3D

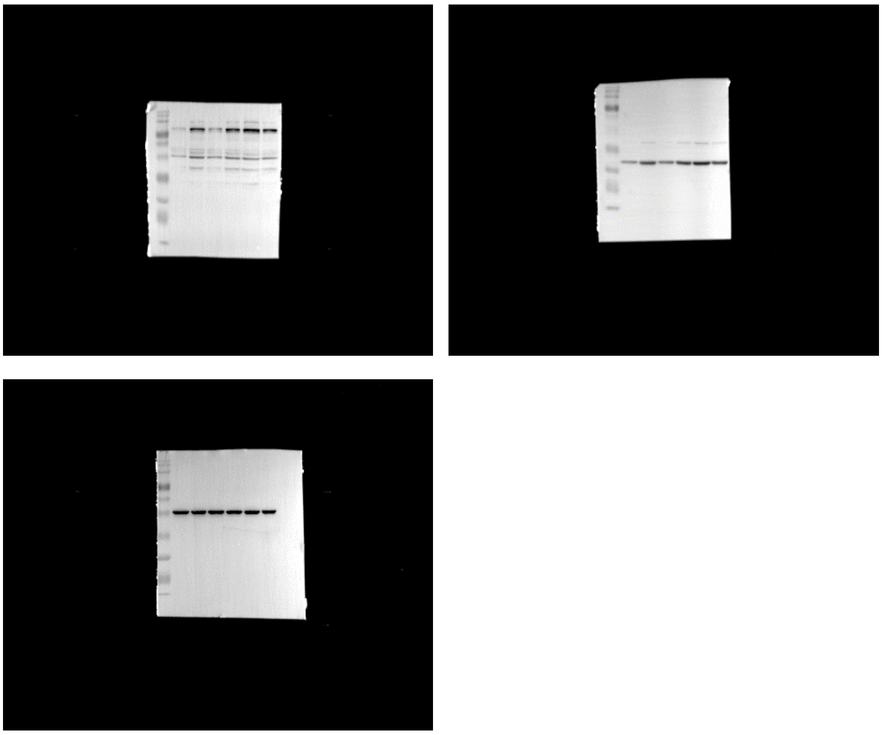

The full uncropped Gels and Blots images of HIF-1α, VEGF and β-actin for Fig 4C

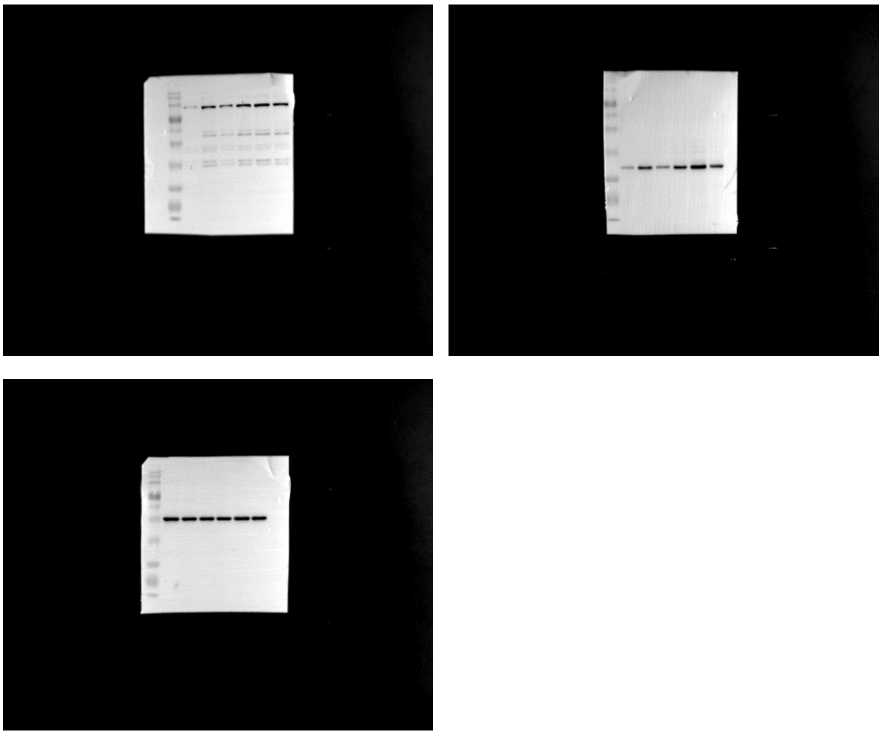

The full uncropped Gels and Blots images of HIF-1α, VEGF and β-actin for Fig 4E
