# Supplementary material for: The role and targeting potential analysis of angiogenesis-related target THY1 in DSS-induced acute colitis in mice
Source: PLoS One. 2026 Jun 2;21(6):e0350385. doi: 10.1371/journal.pone.0350385 (PMC13229366; doi:10.1371/journal.pone.0350385)
Supplement: Attachment — Submitted filename: Response to reviewers.docx [file pone.0350385.s006.docx]

**PONE-D-26-02176**

The role and targeting potential analysis of angiogenesis-related target THY1 in DSS-induced acute colitis in mice

**PLOS One**

Dear editor,

Thank you very much for your letter enclosing the academic editor and reviewers’ comments. We appreciate the academic editor and reviewers’ comments for further improving our manuscript PONE-D-26-02176. Those comments are all valuable and very helpful for revising and improving our paper. We have carefully reviewed the comments and have revised the manuscript accordingly, which we hope meet with approval. In the following pages are our point-by-point responses or a rebuttal against each point raised by the academic editor and the reviewers.

We shall look forward to hearing from you at your earliest convenience.

With regards,

Yingjian Zhang

**Responses to the academic editor and reviewers’ comments:**

Journal Requirements:

When submitting your revision, we need you to address these additional requirements.

1. Please ensure that your manuscript meets PLOS ONE's style requirements, including those for file naming. The PLOS ONE style templates can be found at

https://journals.plos.org/plosone/s/file?id=wjVg/PLOSOne_formatting_sample_main_body.pdf and

<https://journals.plos.org/plosone/s/file?id=ba62/PLOSOne_formatting_sample_title_authors_affiliations.pdf>

**Response:** We have carefully revised our manuscript body formatting according to the PLOS ONE style templates. Manuscript Organization, heading style and File Naming for Figures have been proofread to meets PLOS ONE's style requirements.

2. Your ethics statement should only appear in the Methods section of your manuscript. If your ethics statement is written in any section besides the Methods, please delete it from any other section.

**Response:** We have deleted the "Ethical Considerations" section from our manuscript according to the Journal Requirement.

3. PLOS ONE now requires that authors provide the original uncropped and unadjusted images underlying all blot or gel results reported in a submission’s figures or Supporting Information files. This policy and the journal’s other requirements for blot/gel reporting and figure preparation are described in detail at https://journals.plos.org/plosone/s/figures#loc-blot-and-gel-reporting-requirements and https://journals.plos.org/plosone/s/figures#loc-preparing-figures-from-image-files. When you submit your revised manuscript, please ensure that your figures adhere fully to these guidelines and provide the original underlying images for all blot or gel data reported in your submission. See the following link for instructions on providing the original image data: https://journals.plos.org/plosone/s/figures#loc-original-images-for-blots-and-gels.

**Response:** The full uncropped Gels and Blots images used in this study have been provided as S3 Fig in Supporting Information.

In your cover letter, please note whether your blot/gel image data are in Supporting Information or posted at a public data repository, provide the repository URL if relevant, and provide specific details as to which raw blot/gel images, if any, are not available. Email us at plosone@plos.org if you have any questions.

**Response:** Checked.

4. Please include captions for your Supporting Information files at the end of your manuscript, and update any in-text citations to match accordingly. Please see our Supporting Information guidelines for more information: <http://journals.plos.org/plosone/s/supporting-information>.

**Response:** We have included captions for our Supporting Information files at the end of the manuscript, and updated any in-text citations to match accordingly.

5. If the reviewer comments include a recommendation to cite specific previously published works, please review and evaluate these publications to determine whether they are relevant and should be cited. There is no requirement to cite these works unless the editor has indicated otherwise.

**Response:** Checked.

Reviewer #1: The manuscript entitled “The role and targeting potential analysis of angiogenesis-related target THY1 in DSS-
induced acute colitis in mice” explores the role of THY1 in two aspects of DSS-induced colitis: alterations in colonocyte biology and changes in macrophage phenotypes. While the study apparently focuses on how THY1 regulates the phenotypic alterations of these cells that lead to colitis pathology, there are specific points which are not clear to me in terms of logic, as outlined below.

Major points

1- How well-established is the cell line-based model of IBD (lines 159-161)? Authors need to cite references.

**Response:** We are very appreciating the reviewer’s comments. According to the reviewer’s suggestion, we have added the relevant reference (<https://doi.org/10.5114/ceji.2025.149579>) to clarify the maturity of the colitis cell model in our study. According to cited reference, colitis cell models were constructed by treating human normal colonic epithelial cells (FHC) with different concentrations of lipopolysaccharide (LPS) (1, 3, 5, 10, and 15 ng/ml). In our study, normal human intestinal epithelial cell line NCM460 were gradient intervention with different concentrations of dextran sulfate sodium (DSS) (e.g., 0, 0.1, 0.2, 0.5, 1, 2 μg/mL) for 12 hours to establish an IBD cell model. And the expression levels of inflammatory factors including IL-1β, IL-6, TNF-α, and TGF-β in cells were determined to confirm the successful establishment of the model. Our results indicate that an inflammatory bowel disease (IBD) cell model was successfully established based on this cell line.

2- How could the authors guarantee that the lentiviral vector-mediated delivery of the constructs could solely be targeted to colonocytes and not any stromal or immune cells around them? This is particularly important as THY1 expression on stromal cells has a crucial role in colitis pathogenesis (reference: <https://doi.org/10.4049/jimmunol.212.supp.1326.5032>).

**Response:** We are very appreciating the reviewer’s comments. We cannot guarantee that the lentiviral vector-mediated delivery of the constructs only targets colonic cells without targeting any surrounding stromal cells or immune cells. However, our study aims to evaluate the role of THY1 in the pathogenesis of DSS-induced colitis in mice, rather than the role of THY1 expression in a single cell type. Therefore, we used lentiviral vector-mediated construct delivery to regulate the expression of THY1 in the colonic tissue of mice, as shown in the results of our Fig 1B.

3- The 2nd point of the results (lines 303-348) only shows that THY1 causes definite changes in macrophage phenotypes parallel to the aggravation of DSS-induced colitis in mice. How could the authors draw a causal inference in the statement “THY1 promotes the pathological process of DSS-induced colitis through inhibiting M2 macrophage polarization?” To establish such causal connection, authors need to experimentally deplete macrophages or alter macrophage phenotypic states under THY1-OE or THY1-silenced conditions, and repeat the same experiments in vivo.

**Response:** We are very appreciating the reviewer’s comments. We acknowledge that it is necessary to add the experiments suggested by the reviewer to draw the causal inference that "THY1 promotes the pathological process of DSS-induced colitis through inhibiting M2 macrophage polarization." Therefore, we have changed the second point of the results to "THY1 promotes the pathological process of DSS-induced colitis and inhibits M2 macrophage polarization."

4- In the same point, the authors seemingly check several different aspects (cytokines, ROS, angiogenesis markers) related to macrophages. However, there is no definitive proof that the changes in these markers due to THY1 manipulations are solely, or at least majorly, related to their altered output from macrophages in vivo. In addition, why they check so many aspects together without any apparently clear hypothesis is not understandable.

**Response:** We are very appreciating the reviewer’s comments. The detection of cytokines, reactive oxygen species, and angiogenesis markers is to clarify the effect of THY1 on the pathological process of DSS-induced colitis. First, we found the regulatory effect of THY1 on cytokines, reactive oxygen species, and angiogenesis markers through in vivo experiments. Given that macrophages play an important immunomodulatory role in the pathological process of colitis, in this study, we further elucidated the molecular mechanism from the perspective of macrophage immunomodulation through an in vitro co-culture system of intestinal epithelial cells and macrophages.

5- Again, in the coming sections, authors assess colonocyte apoptosis and angiogenesis marker levels without any clear connection to the previous sections. The entire story looks like an ensemble of experiments without any proper logical link among them.

**Response:** We are very appreciating the reviewer’s comments. This study aimed to explored and demonstrated the potential role of THY1 in the pathological mechanism of IBD from multiple perspectives based on DSS-induced acute colitis in mice and colonocytes models. Existing studies have shown that the impairment of the intestinal epithelial cell barrier function caused by colonic cell apoptosis is a key initiating factor in the pathogenesis of IBD, and abnormal angiogenesis can provide nutritional support for the infiltration of inflammatory cells and exacerbate the inflammatory response. Therefore, on the basis of revealing the potential role of THY1 in the pathological mechanism of IBD from the perspective of macrophage immunomodulation, we further revealed the role of THY1 in the pathogenesis of IBD from the perspectives of colonocyte apoptosis and abnormal angiogenesis, aiming to provide theoretical support for the development of THY1-targeted IBD treatment strategies. We acknowledge that the proper logical link among them was not shown in this study. However, this study has completely revealed the role and targeting potential of angiogenesis-related target THY1 in DSS-induced acute colitis in mice. Moreover, we have pointed out the limitations of this study in the discussion section.

6- Although the authors check multiple phenotypic effects of THY1 manipulations on colonocytes, how each of them affects colitis has not been checked. So the entire set of findings by large stays correlative.

**Response:** We are very appreciating the reviewer’s comments. Regarding the limitations of this study pointed out by the reviewer, it should be noted that in this study, we aimed to explored and demonstrated the potential role of THY1 in the pathological mechanism of IBD from multiple perspectives based on DSS-induced acute colitis in mice and colonocytes models, providing theoretical support for the development of THY1-targeted IBD treatment strategies. In addition, in the discussion section, we also pointed out the limitations of this study, including that the molecular mechanism by which THY1 mediates angiogenesis and affects colitis needs to be fully elucidated.

7- Why do the authors check ROS-related phenotypes (ROS, MDA, GSH etc) in cell supernatants? It would have been more physiologically relevant if they had done the experiment with cell homogenates/lysates.

**Response:** We are very appreciating the reviewer’s comments. We already have done the experiment with cell homogenates/lysates, which was shown as S3 in Supporting Information files. According to the review’s comments, we have replaced the detection results in the cell supernatant with those in the cell homogenate/lysate, as revised in Fig 2B.

8- None of the experiments involve DSS-conditioning of the THP-1 cells, and only involves co-culturing them with colonocytes of different phenotypes. This violates the physiology of colitis, where macrophages also encounter DSS, and are likely affected by it to some extent as well (reference: https://doi.org/10.1691/ph.2016.6688).

**Response:** We are very appreciating the reviewer’s comments. In our co-culture system of intestinal epithelial cells and macrophages, we aimed to investigate the immunomodulatory role of macrophages in the pathological process of colitis regulated by THY1. Therefore, the DSS-conditioning of the THP-1 cells group was not necessary.

Minor points

1- It would have been preferable if the authors would have shown the time kinetics of THY1 expression in colon along the course of DSS treatment in mice and afterwards.

**Response:** We are very appreciating the reviewer’s comments. In this study, we used lentiviral vector-mediated construct delivery to regulate the expression of THY1 in the colonic tissue of mice. Through end-point mode evaluation, we further explored the potential role of THY1 in the pathological mechanism of DSS-induced colitis. It is worth noting that the temporal dynamics of THY1 expression in colonic tissues were not the core presentation content of this study, so they were not presented in detail.

2- For apoptosis assay, how could the authors resuspend the cells in 1 microliter binding buffer? I suspect it is a typing error, and needs to be taken care of. Furthermore, didn’t they use any strategy for gating live/dead cells differentially in their flow cytometry data for macrophages?

**Response:** We are very appreciating the reviewer’s comments. By examining the data from our apoptosis assay, we found that the cells were resuspended in 300 microliters of binding buffer. Therefore, we have revised the error in text as follows: “Cells were harvested, rinsed with PBS, and resuspended in 300 microliters of Annexin V binding buffer to achieve a single-cell suspension.” For macrophage immunophenotyping analysis, we adopted a gating strategy to select live CD11b+ macrophages. Specifically, we first excluded debris and doublet cells using forward scatter (FSC)/side scatter (SSC) parameters, then selected live CD11b+ monocytes/macrophages. We have added the following modification to the text: “The gating strategy adopted a step-by-step approach: First, use the forward scatter (FSC)/side scatter (SSC) parameters to exclude debris and doublet cells, and then select live CD11b+ monocytes/macrophages.”

3- If colonocytes were the target, why did the authors opt for lentivirus injection intravenously?

**Response:** We are very appreciating the reviewer’s comments. Our study aims to evaluate the role of THY1 in the pathogenesis of DSS-induced colitis in mice, rather than the role of THY1 expression in a single cell type. Therefore, we used lentiviral vector-mediated construct delivery to regulate the expression of THY1 in the colonic tissue of mice, as shown in the results of our Fig 1B.

4- Why didn't the authors opt for showing the intestines from mice groups photographically? It might have been more convincing than putting up a graph.

**Response:** We are very appreciating the reviewer’s comments. Since all groups of colons were not arranged and photographed during the mouse dissection, we did not visually present pictures of intestinal tissues. However, we have taken comparative photos of the THY1 overexpression group and the Sham group, as shown below. We guarantee that all data is real and reliable, and no photo display is required.

5- A much better way to represent the data in fig. 3 would have been to gate on the CD11b+ cells, followed by the assessment of CD86 and CD206 simultaneously in them.

**Response:** We are very appreciating the reviewer’s comments. In this study, we have adopted a gating strategy to select live CD11b+ macrophages for macrophage immunophenotyping analysis. According to the review’s second minor point, we have added the following modification to the method section: “The gating strategy adopted a step-by-step approach: First, use the forward scatter (FSC)/side scatter (SSC) parameters to exclude debris and doublet cells, and then select live CD11b+ monocytes/macrophages.” In addition, given the large number of groups included in this study, incorporating the gating data into the current Fig 3 would result in an excessive number of panels within the overall data graph. This would compromise the clarity and effectiveness of the presentation. Based on this, we believe that there is no need to add additional gating data at present to ensure the simplicity of the chart and the effectiveness of information transmission.

6- For cleaved caspase-3 immunoblots, total caspase-3 level needs to be demonstrated as well.

**Response:** We are very appreciating the reviewer’s comments. We acknowledge that the total caspase3- level should be presented simultaneously to improve the rigor and interpretability of the experiment. We deeply apologize for the oversight in not presenting the total caspase-3 level simultaneously in the initial submission. Nevertheless, it is important to note that the Western blot results of cleaved caspase-3 clearly showed two bands, indicating the high accuracy and reliability of our experimental procedures.

7- Was VEGF levels quantified with or without Golgi-stop/Golgi-plug treatment?

**Response:** We are very appreciating the reviewer’s comments. In this study, VEGF levels were quantified without Golgi-stop/Golgi-plug treatment.

8- For mice experiments, plotting individual data points is always preferable. In addition, it is better to plot SEM rather than SD for showing data corresponding to biological replicates.

**Response:** We are very appreciating the reviewer’s comments. In this study, the number of biological replicates was set to 6, and the data were verified to conform to a normal distribution. Therefore, using Mean ± SD (mean ± standard deviation) as a descriptive statistical indicator is a reasonable choice. In contrast, although Mean ± SEM (mean ± standard error) is often used to infer the confidence interval of the population mean and highlight the "accuracy" or "reliability" of the results, its applicability is more dependent on a larger sample size. Since the sample size in this study is relatively limited, using Mean ± SEM may mask the variability of the data, thus weakening the intuitive reflection of the actual data distribution. Therefore, under the current experimental conditions, Mean ± SD can more accurately present the characteristics and distribution of the data.

9- Did the authors perform normality tests before doing parametric statistical tests?

**Response:** We are very appreciating the reviewer’s comments. We confirm that normality tests were conducted before performing the parametric statistical tests.

10- In the cases where authors have claimed “THY1 does so..” and on, should be reframed as “colonocyte-expressed THY1 does so…” or “THY1 on colonocytes does so….”. This will specify the findings.

**Response:** We are very appreciating the reviewer’s comments. According to the review’s suggestion, we have reframed “THY1 does so” as “THY1 on colonocytes does so…” in cell experiments to specify the findings.

Reviewer #2: The study by Pengliang Zhang et al claim to identify a novel role of THY 1 in DSS induced colitis. They found out that THY1 promotes inflammatory angiogenesis that exacerbate the disease. The methods and models employed enrich the study and the data gathered collectively reinforces the key hypothesis of the study. The hard work is commendable. However some clarifications are needed.

1.) The study doesn’t appear to be unique and the first of it’s kind as evident from the sentence “In this study, we first revealed the crucial role of the angiogenesis-related target THY1 in the development of acute colitis”. One similar study “The Journal of Immunology, Volume 212, Issue 1,1326_5032, https://doi.org/10.4049/jimmunol.212.supp.1326.5032” published in 2024 had a similar objective and conclusion. Many more can be cited. Hence, it appears that the authors reinforces and substantiate the earlier studies.

**Response:** We are very appreciating the reviewer’s comments. According to the review’s comments, we have revised the sentence to “In this study, we revealed the crucial role of the angiogenesis-related target THY1 in the development of acute colitis”.

2.) Use of a variety of techniques and models often overwhelm the readers. The design could have been much simplified and succinctly presented to make reading and assimilation easier.

**Response:** We are very appreciating the reviewer’s comments. According to the review’s comments, we have presented the content in a more concise way to enhance the readability of the text and the convenience of understanding.

3.) Although the authors state the use of AI for improving English, the English still need ample revision.

**Response:** We are very appreciating the reviewer’s comments. According to the review’s comments, we have carefully checked and corrected the expression errors in the text. At the same time, we further optimized the language expression to make the overall writing more fluent and accurate.

4.) In the co-culture experiment, choice of human cell lines NCM460 and THP-1 is not accurate. A co-culture of Mice primary intentional epithelial cells and peritoneal macrophages would have been more appropriate to establish and validate the in vitro model

**Response:** We are very appreciating the reviewer’s comments. Co-culturing primary mice primary intentional epithelial cells and peritoneal macrophages can indeed more precisely reveal the function and molecular mechanism of THY1 in the mouse model of IBD. However, the main objective of this study is to comprehensively evaluate the functional role of THY1 in IBD through animal models and cell models. Therefore, in the co-culture experiment, we selected the human cell lines NCM460 and THP-1 as standardized experimental models. This choice not only facilitates experimental operations but also ensures the reproducibility and reliability of the research results.

5.) What is the natural ligand for THY1 and are there any inhibitors of THY1 that may have been used alongside or instead of sh-THY1?

**Response:** We are very appreciating the reviewer’s comments. In this study, we aimed to explored and demonstrated the potential role of THY1 in the pathological mechanism of IBD from multiple perspectives based on DSS-induced acute colitis in mice and colonocytes models, providing theoretical support for the development of THY1-targeted IBD treatment strategies. We believe that the application of THY1 inhibitors should be based on a deep understanding of the regulatory functions of THY1. Based on this, we adopted a lentiviral vector-mediated construct delivery strategy to achieve precise regulation of THY1 expression levels in mouse colon tissues and intestinal epithelial cells.

The study presents a detailed evaluation of the effect of THY1 in colitis, which needs substantial refinement for enhanced clarity in light of the points raised.

**Response:** We are very appreciating the reviewer’s comments. Regarding the limitations of this study pointed out by the reviewer, it should be noted that in the discussion section, we have pointed out the limitations of this study, including that the molecular mechanism by which THY1 mediates angiogenesis and affects colitis needs to be fully elucidated.
